# Endothelial Cell Processing and Alternatively Spliced Transcripts of Factor VIII: Potential Implications for Coagulation Cascades and Pulmonary Hypertension

**DOI:** 10.1371/journal.pone.0009154

**Published:** 2010-02-11

**Authors:** Claire L. Shovlin, Gillian Angus, Richard A. Manning, Grace N. Okoli, Fatima S. Govani, Kay Elderfield, Graeme M. Birdsey, Inês G. Mollet, Michael A. Laffan, Francesco A. Mauri

**Affiliations:** 1 National Heart and Lung Institute Cardiovascular Sciences, Imperial College London, London, United Kingdom; 2 Respiratory Medicine, Imperial College Healthcare National Health Services Trust, London, United Kingdom; 3 Investigative Sciences, Imperial College London, London, United Kingdom; 4 Haematology, Imperial College Healthcare National Health Services Trust, London, United Kingdom; 5 Histopathology, Imperial College Healthcare NHS Trust, London, United Kingdom; 6 Institute of Molecular Medicine, University of Lisbon, Lisbon, Portugal; Oregon Health and Science University, United States of America

## Abstract

**Background:**

Coagulation factor VIII (FVIII) deficiency leads to haemophilia A. Conversely, elevated plasma levels are a strong predictor of recurrent venous thromboemboli and pulmonary hypertension phenotypes in which in situ thromboses are implicated. Extrahepatic sources of plasma FVIII are implicated, but have remained elusive.

**Methodology/Principal Findings:**

Immunohistochemistry of normal human lung tissue, and confocal microscopy, flow cytometry, and ELISA quantification of conditioned media from normal primary endothelial cells were used to examine endothelial expression of FVIII and coexpression with von Willebrand Factor (vWF), which protects secreted FVIII heavy chain from rapid proteloysis. FVIII transcripts predicted from database mining were identified by rt-PCR and sequencing. FVIII mAb-reactive material was demonstrated in CD31+ endothelial cells in normal human lung tissue, and in primary pulmonary artery, pulmonary microvascular, and dermal microvascular endothelial cells. In pulmonary endothelial cells, this protein occasionally colocalized with vWF, centered on Weibel Palade bodies. Pulmonary artery and pulmonary microvascular endothelial cells secreted low levels of FVIII and vWF to conditioned media, and demonstrated cell surface expression of FVIII and vWF Ab–reacting proteins compared to an isotype control. Four endothelial splice isoforms were identified. Two utilize transcription start sites in alternate 5′ exons within the *int22h-1* repeat responsible for intron 22 inversions in 40% of severe haemophiliacs. A reciprocal relationship between the presence of short isoforms and full-length FVIII transcript suggested potential splice-switching mechanisms.

**Conclusions/Significance:**

The pulmonary endothelium is confirmed as a site of FVIII secretion, with evidence of synthesis, cell surface expression, and coexpression with vWF. There is complex alternate transcription initiation from the FVIII gene. These findings provide a framework for future research on the regulation and perturbation of FVIII synthesis, and of potential relevance to haemophilia, thromboses, and pulmonary hypertensive states.

## Introduction

Coagulation cascade activation is essential for normal haemostasis [1]. Activated factor VIII (FVIIIa) is responsible for sustained intravascular generation of thrombin via its role as a cofactor for FIXa in the intrinsic Xase, with FVIIIa/FIXa ultimately responsible for most of the FXa produced by both extrinsic (tissue-factor initiated) and intrinsic coagulation cascades [2]. FVIII deficiency leads to the bleeding disorder haemophilia A (OMIM +306700) [3].

Conversely, elevated levels of FVIII are emerging as one of the strongest predictors of recurrent venous thromboembolic events [4], [5]. Venous thromboemboli (deep venous thromboses and pulmonary emboli) carry significant health burdens [6], including the development of chronic thromboembolic pulmonary hypertension [7] in up to 3.8% of cases of pulmonary emboli at 2 year follow up [8]. Elevated plasma levels of FVIII are unusual amongst general thrombotic risk factors, as they are not only a risk factor for venous thromboembolism, but also associated with chronic thromboembolic pulmonary hypertension [9], [10]. High levels of von Willebrand Factor (vWF), the glycoprotein with which FVIII circulates in a non-covalent complex [11], are also observed in pulmonary hypertensive states [9], [10].

The liver produces sufficient FVIII for normal plasma levels [12], with immunohistochemical evidence for stronger expression by hepatic sinusoidal endothelial cells than hepatocytes [13]. Extrahepatic sources can also contribute to circulating levels of FVIII, as demonstrated by the surprisingly high residual FVIII plasma levels in dogs transplanted with haemophiliac livers [14].

We hypothesised that pulmonary endothelial cells might be a source of plasma FVIII after demonstrating an age-independent association between elevated plasma FVIII levels and pulmonary arteriovenous malformations (AVMs) in hereditary haemorrhagic telangiectasia (HHT) [15]. A pulmonary endothelial source of FVIII would be of particular importance to these patients whose ischaemic strokes are attributed to paradoxical thromboemboli through pulmonary AVMs [16], and who are also at risk of several pulmonary forms of pulmonary hypertension [17]. Pulmonary endothelial synthesis of FVIII would also be of importance to patients with *in situ* pulmonary thromboses, and potentially, of immense importance to the haemostatic balance, since pulmonary endothelial cells provide an endothelial-blood interface approximately twenty times all other vessels combined [18].

Others have demonstrated accumulation of FVIII in the effluent from an isolated reperfusion model of lungs from three of four heart-beating donors, and conditioned medium of early passage pulmonary microvascular endothelial cells [19]. In this study, we examined expression and processing of FVIII by a number of normal pulmonary and systemic endothelial types, *in situ* and *in vitro*, and compared to expression of von Willebrand Factor.

## Materials and Methods

### Subjects/Source of Endothelial Cells

a) Normal primary endothelial cells: Human pulmonary artery endothelial cells (HPAEC), pulmonary microvascular EC (HPMEC) and human dermal microvascular EC (HDMEC) were purchased from PromoCell GmbH, Heidelberg, Germany. All Figures are from lot numbers HPAEC 5060806.7, 5110901.3 and 6031391.3; HPMEC 7042502.7. Three lots of locally derived human umbilical vein EC (HUVEC), derived from anonymised and untraceable human umbilical cords, as specifically approved by the Hammersmith Hospitals Research Ethics Committee (ref no. 06/Q0406/21), were also studied. All EC were from separate donors.

b) Human lung tissue: Frozen normal human lung tissue was obtained from the Human Biomaterials Resource Centre of the Hammersmith Hospitals Trust, with ethical approval from the MREC for Wales (07/MRE09/54). In this protocol, written consent for the use of waste material for research was part of the procedural consent for the operation. This is obtained prior to the procedure, and filed in the patient's healthcare records. All tissue handling was compliant with the requirements of the Human Tissue Act (2004). Coded material was supplied accompanied by a minimum data set (tissue type, pathology and clinical diagnosis together with the age (in years) and ethnic group of the donor). Five separate donors were examined.

### Primary Antibodies

**Figure 1:**
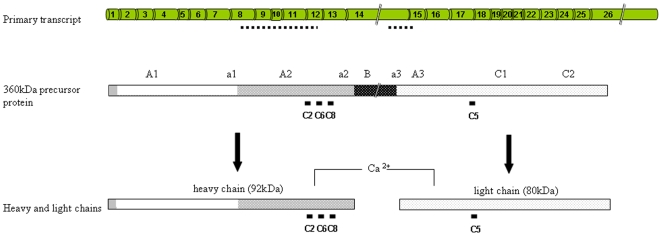
FVIII genomic and domain structure. FVIII undergoes a complex series of steps between primary transcript synthesis and eventual activity [20]. The 9 kb ‘full length’ 26 exon primary mRNA transcript is translated to a 360 kDa polypeptide chain which is translocated to the ER and Golgi for post-translational processing including B domain proteolysis to generate the mature heavy and light chains [21], [22]. Bars indicate the epitope sites for mAbs C2, C5, C6 and C8 which react with the 360 kDa precursor, and the heavy (C2, C6, C8) or light chains (C5) [23]. Dotted lines indicate the sites of previously described transcriptional silencing regions (see text).

FVIII expression was primarily examined using a series of hybridoma-derived murine anti-human FVIII:Ag IgG_1_ κ monoclonal antibodies (C2, C5, C6 and C8 mAbs; Figure 1) which display no vWF cross reactivity [23] and were used to purify FVIII for the original Genentech cloning. FVIII ELISA experiments (see below) utilised monoclonal antibody ESH-4 coated to the microtitre wells as the capture antibody of the Imunobind FVIII ELISA (Axis Shield, Bicton, UK, #ADI-884CON). Other primary antibodies were obtained from DAKO UK Ltd, Ely, UK (monoclonal mouse IgG_1_ antibody to *Aspergillus niger* glucose oxidase (X0931) as an isotype control; anti-human CD31 clone JC70A (M0823); and rabbit anti-human VWF polyclonal Ab (A0082)).

### Immunohistochemistry

Serial sections from blocks from the five separate donors were immunostained for 1 hour at room temperature with control mouse IgG_1_ (20 µg/ml), anti-CD31 mAb (20 µg/ml), or FVIII C5 mAb (20 µg/ml). Stained sections were incubated with biotinylated goat anti-mouse IgG (DAKO UK Ltd, Ely, UK) followed by a Streptavidin alkaline phosphatase-conjugate (Roche Diagnostics GmbH, Mannheim, Germany). Fast Red Kit (BioGenex, San Ramon CA, USA) development was followed by Mayer haematoxylin counterstaining.

### Endothelial Cell Cultures

Human pulmonary artery endothelial cells (HPAEC), pulmonary microvascular EC (HPMEC) and human dermal microvascular EC (HDMEC) were from PromoCell GmbH, Heidelberg, Germany. These primary human endothelial cells (not cell lines) were ordered to arrive at passage two in a proliferating state: On arrival, after inspection for confluency and viability, they were rested for 2 hr at 37°C, 5% CO_2_, before the addition of fresh reconstituted Promocell EC or microvascular EC (MEC) media (2% and 5% fetal calf serum respectively), and subsequent passage according to degree of confluency and experimental design. Experiments were performed within 10–15 doubling times as recommended. All Figures are from EC at passage 4–6.

### Confocal Microscopy

EC were seeded at 3×10^5^ in individual wells of BD Falcon CultureSlides (354118, BD Biosciences, Oxford, UK). After 24–48 hr, media was either left in situ (unstimulated cells) or replaced with fresh media containing 0.5 U/ml thrombin (T4393, Sigma Aldrich, Gillingham, UK) for 6 hours. Following phosphate buffered saline (PBS) washes, cells were fixed and permeabilised with methanol at −20°C for at least 10 minutes. After methanol aspiration, and two further PBS washes, cells were incubated with 100 µl of primary antibody (diluted to 1 ug/ml in 3% bovine serum albumin (BSA) for the four mouse anti-FVIII mAbs; 3 µg/ml for rabbit anti-vWF pAb), or control media for 15 minutes at room temperature. Cells were washed in PBS, then incubated with 150 µl of appropriate fluorescently conjugated secondary antibodies (Alexa Fluor 488 goat anti-rabbit IgG or Alexa Fluor 555 goat anti-mouse IgG) in 3% BSA, with or without Alexa Fluor 633 To-Pro-3 for nuclear counterstain (all from Invitrogen, Paisley, UK). Following incubation in the dark for 15 minutes, aspiration, and PBS washes, the CultureSlide was disassembled, stained cells mounted in anti-fade medium (Vectashield®, Vector Laboratories, Burlingame, CA, USA), and imaged following no more than 3–4 days storage in the dark at 4°C. Images were acquired using the Plan-Neofluor 20x/0.5 and 40x/1.30 objectives of an LSM 510 META inverted fluorescence confocal microscope (Carl Zeiss, Welwyn Garden City, UK). Each channel was collected sequentially using LSM Image Browser software. In preliminary experiments in HPAEC, all four FVIII mAbs demonstrated comparable staining patterns.

### ELISAs

EC were seeded into 24 well plates and cultured to confluency. 500 µl of fresh media (+/−0.5 U/ml thrombin (T4393, Sigma Aldrich, Gillingham, UK)) was added, with at least three replicates for each condition, donor or cell type. Conditioned medium was aspirated from triplicate wells after 24 hr and 48 hr for immediate storage in aliquots at −70°C. Reconstituted control media of each type was also dispensed to 3 separate wells of a 6 well plate and incubated at 37°C, 5% CO_2_ for 24 hours prior to immediate storage in aliquots at −70°C. FVIII:Ag was detected using a commercial FVIII ELISA (Immunobind ADI-884CON, Axis Shield, Bicton, UK), with standard curves generated using supplied lyophilised FVIII concentrates. VWF:Ag was detected as previously described [24]. All samples were processed in duplicate. Optical readouts were quantified on an Opsys MR Plate Reader using Revelation Quicklink Software (both from Dynex Technologies, Worthing, UK). All data sets were included in the final analyses.

### FVIII Activity Assessments

Initial FVIII activity measurements were performed by running diluted conditioned media through a CA7000 automated coagulation analyser (Sysmex UK, Milton Keynes, UK) in the hospital service laboratory. Samples were also analysed undiluted in a manual one-stage assay in which 0.1 mls of congenital FVIII deficient plasma (Technoclone Ltd, Dorking, UK), and 0.1 mls of test plasma or media, were aliquoted into glass tubes, and a stop watch started immediately after the addition to the tubes of 0.1 mls of APTT reagent (Actin FS, Dade, Sysmex UK Ltd, Milton Keynes, UK). The time to the generation of a clot was recorded.

### Flow Cytometry

EC were seeded into 12 well plate wells and allowed to reach confluency. Triplicate confluent samples of each HPAEC, HPMEC and HUVEC were trypsinised, washed in Hanks buffered saline solution (HBSS), and aliquoted into centrifuge tubes for sedimentation, prior to resuspension in 50 µl residual media, and addition of 50 µl HBSS and 1% fetal calf serum (FCS) +/− 1 µg murine FVIII mAb C2, rabbit anti-vWF, or control IgG_1_ for 20 minutes at 4°C. After washing and centrifugation, all cells were resuspended in 50 µl residual media, and treated with 50 µl HBSS/1% FCS containing 1 µg of an appropriate secondary conjugated antibody (Alexa Fluor 488 goat anti-mouse IgG for FVIII and controls; Alexa Fluor 488 goat anti-rabbit IgG for vWF). Cells were incubated in the dark for 20 minutes at 4°C, prior to a further wash, PBS resuspension, and immediate analysis using an EPICS XL flow cytometer (Beckman Coulter, High Wycombe, UK). Relative fluorescent intensity (RFI) was calculated by dividing the mean fluorescent intensity of test antibody by the fluorescent intensity of untreated or isotype control treated cells from the same well. Hence no expression has an RFI of 1.00.

### FVIII Transcript Analyses

RNA was extracted from EC at various degrees of confluency using Tri® Reagent (Sigma). Following quantification by optical densitometry, 1 µg of RNA was used for cDNA synthesis using Superscript™ II (Invitrogen, Paisley, UK). Oligonucleotide primers were designed to constitutive and alternative exons of the known FVIII transcripts (RefSeq [25] variants 1; NM_000132 and 2; NM_019863), and to novel exons identified by the ExonMine project [26] through extensive mining of BLAT alignments [27], [28], [29] to human genome NCBI Build 36.1 of mRNA and expressed sequence tag (EST) sequences deposited in the GenBank [30]. Rt-PCR products were purified by gel excision or treated with an ExoSAP mix comprising 1.2 u of ExoI and 1.06 u of SAP (Promega, Southampton, UK), sequenced on an ABI 3730x1 DNA analyser, and results analysed using FinchTV V1 4.0 software (Geospiza, Seattle, USA). Confirmation of FVIII transcript identity was obtained by Blastn (http://blast.ncbi.nlm.nih.gov/Blast.cgi). Evolutionary conservation of flanking sequences was performed via the UCSC Genome Browser [29].

### Analyses

The main outcome measures were i) endothelial cell reactivity to Abs assessed qualitatively and semi-quantitatively (see below); ii) ELISA quantification of conditioned media reactivity; iii) flow cytometric quantification of cell surface reactivity; and iv) comparison of transcript detectability by single and nested rt-PCR reactions.

For confocal microscopy, FVIII and vWF expression levels per cell were defined semi-quantitatively by examination of at least five separate high power fields. FVIII expression was graded as undetectable, weak and strong cytoplasmic expression. vWF expression was recorded as 0, 10 or >10 Weibel Palade bodies (WPB) per cell, with high level expression defined as >10 WBP per cell. The percentage of cells expressing the different levels was expressed as mean±standard deviation, calculated using GraphPad Prism version 4, GraphPad Software, San Diego, USA.

For ELISA and flow cytometry, no assumptions were made about data distribution, and non-parametric analyses were performed: Multiple group comparisons were performed using the Kruskal-Wallis test on all datasets, with *p* values from post test analyses of the indicated selected groups using Dunn's Multiple Comparison test. Two group comparisons were performed using the Mann Whitney test.

## Results

### FVIII Protein Expression in Lung Tissue

**Figure 2:**
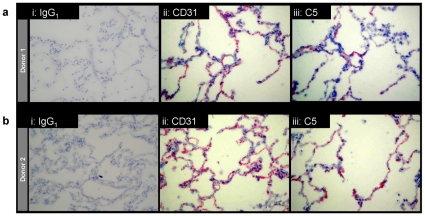
Lung expression of FVIII. Serial sections of frozen normal human lung tissue from two donor blocks (a and b) stained with **i)** control IgG_1_, **ii**) anti-CD31, or **iii**) anti-FVIII (C5). The 200x images are representative of data from all five donors.

Factor VIII expression by the pulmonary endothelium was assessed *in situ*, by comparing serial sections from the same donor blocks stained with the IgG_1_ isotype control mAb, CD31 mAb, or C5 mAb to FVIII. As demonstrated in Figure 2, there was no reactivity to staining using the isotype control antibody (Figure 2ai, Figure 2bi). In contrast, anti-CD31 demarcated the endothelium of pulmonary capillaries in interalveolar septae (Figure 2aii, Figure 2bii), and staining was observed in a similar but less extensive distribution using C5 mAb to FVIII (Figure 2aiii, Figure 2biii). Comparable staining patterns were observed in the other three cases examined.

### FVIII Protein Expression by Endothelial Cells In Vitro

**Figure 3:**
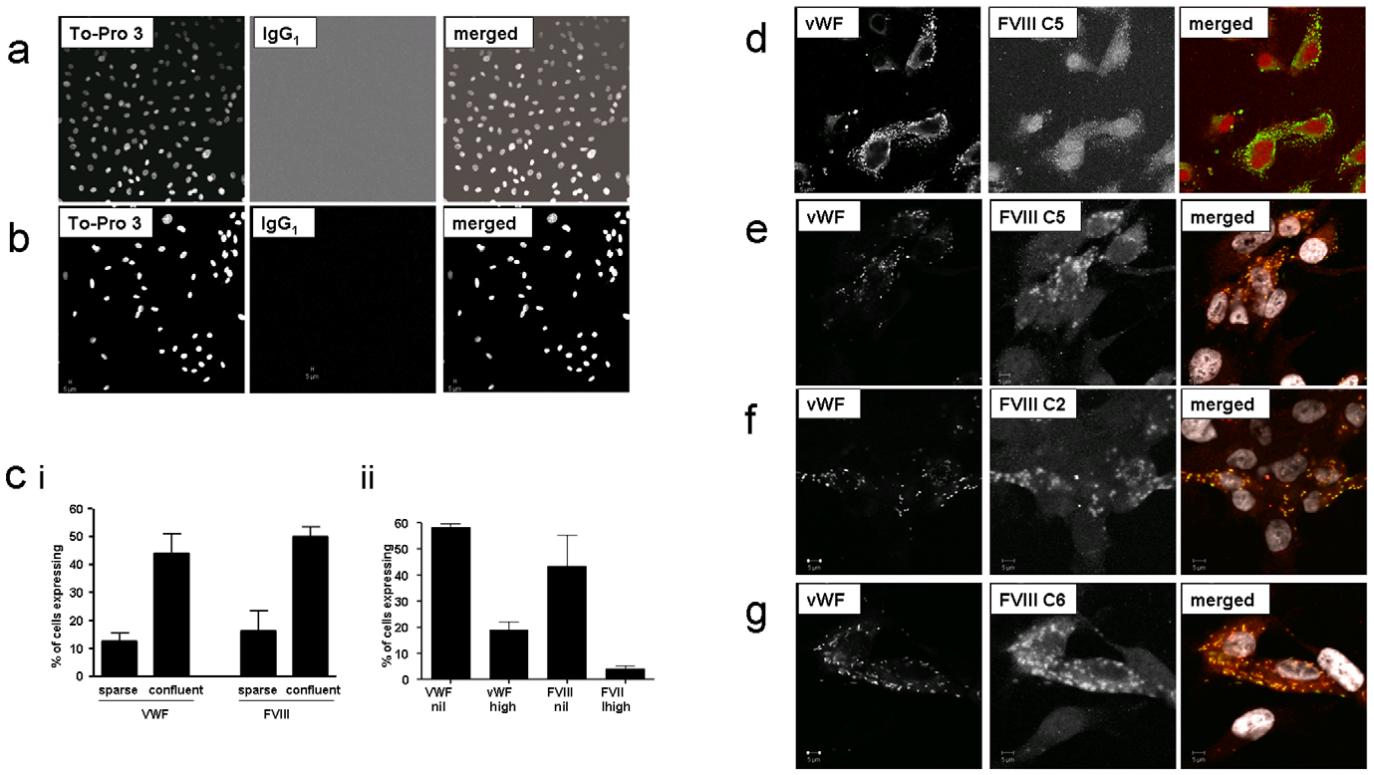
Immunofluorescence images of FVIII expression by EC. **a**: Sequential confocal fluorescence microscopy images in primary human EC. **a** and **b**: Representative HPMEC and HPAEC control images using To-Pro-3 nuclear counterstain (first panel, monochrome), murine control IgG_1_ (second panel monochrome) and merged images (third panel: To-Pro-3 nuclear counterstain white, control IgG_1_ red) using maximum gain used for imaging. **c**) Comparison of proportion of **i**) **HPMEC** and **ii**) **HPAEC** expressing vWF and FVIII protein (expression levels defined in methods). **d, e, f, g**) Sequential confocal fluorescence microscopy images comparing vWF (monochrome in first panel); FVIII (monochrome in second panel, specific mAb as denoted), and merged images (third panel; vWF green, anti-FVIII reacting protein red) in **d**): HPMEC, and **e, f, g**): HPAEC. FVIII mAb images displayed here are representative of all FVIII mAbs examined, and all cell lots. Note yellow merged images suggesting degree of FVIII/vWF colocalisation in **e, f, g**, with white colouring denoting the nuclei (TO-PRO3 nuclear counterstain). Scale bars indicate 5 µm.

Using confocal microscopy, all four mAbs to both heavy (C2, C6, C8) and light (C5) chains demonstrated significant reactivity in HPMEC and HPAEC (Figure 3), HDMEC, but not HUVEC (data not shown). In pulmonary EC, FVIII expression increased with the degree of confluency (Figure 3ci), and for the pulmonary and dermal microvascular EC, with the formation of vessel-like structures *in vitro*. There was no difference in expression patterns following EC treatment with thrombin for 6 hours (data not shown). There was also no difference between appearances for the four antibodies for the same EC lot.

Since vWF is required for stable accumulation of FVIII in cellular supernatants [11], intracellular expression of FVIII and vWF were compared. Anti-vWF defined classical Weibel Palade bodies in a proportion of HPMEC and HPAEC, as in the original description in pulmonary endothelial cells [31]. Expression of VWF also increased with the degree of confluency (Figure 3ci). Overall, a higher proportion of EC expressed vWF than FVIII (Figure 3cii). Usually, high vWF and high FVIII expresssion did not overlap in HPMEC (Figure 3d) or HPAEC. Where coexpression did occur, generally the intracellular sites were non overlapping, but in some HPAEC, colocalisation of FVIII and vWF within the same cells appeared to be centred on vWF-containing Weibel Palade bodies (Figure 3e, Figure 3f, Figure 3g).

### FVIII/vWF Cell Surface Expression

**Figure 4:**
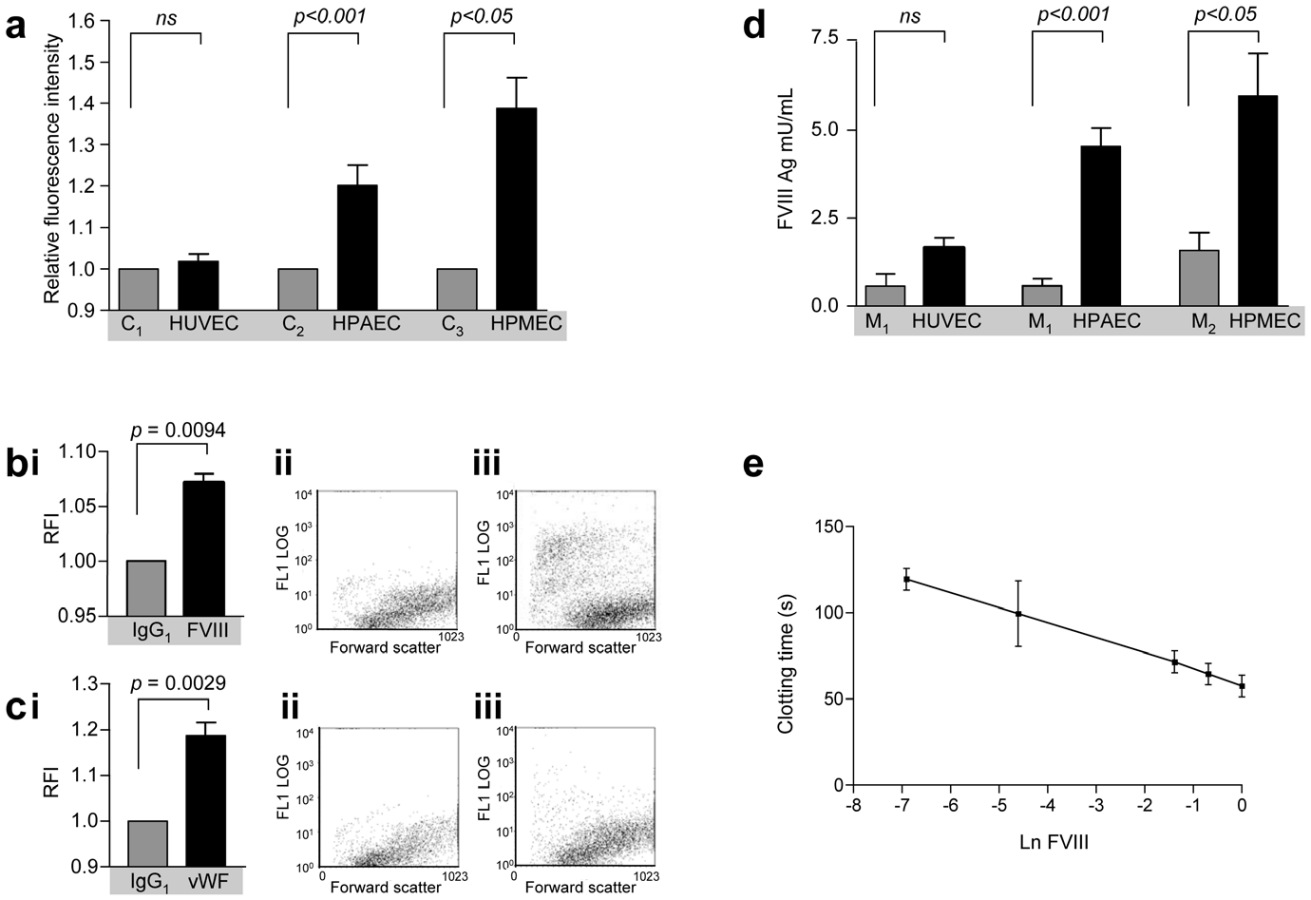
Secreted and cell surface FVIII. **a**) **Quantification of FVIII on the surface of EC.** Relative fluorescence intensity (RFI) of confluent HUVEC, HPAEC and HPMEC stained with FVIII mAb C2, compared to EC from the same well in which C2 was omitted. **b**)** Quantification of HPAEC surface FVIII: bi** Comparison of EC RFI for HPAEC from the same well treated with IgG_1_ control, or C2 mAb; p values calculated by Mann Whitney. **bii-iii**. Representative raw data plots of log expression (FL1 log) versus forward scatter as a marker of cell size for HPAEC from the same well treated with **bii**) IgG_1_ control, **biii**) C2 mAb. **c**)** Quantification of HPAEC surface vWF. ci**) Comparison of EC RFI for HPAEC from the same well treated with IgG_1_ control, or vWF pAb, p values calculated by Mann Whitney. **cii-iii**. Representative raw data plots of log expression (FL1 log) versus forward scatter as a marker of cell size for HPAEC from the same well treated with **cii**) IgG_1_ control, **ciii**) vWF pAb. **d**) **Quantification of FVIII:Ag in control and EC-conditioned media by ELISA**. M_1_ denotes control EC media (2% FCS), M_2_ denotes control MEC media (5% FCS). M_1_ control data are presented twice for clarity. *P* values are presented for the 48 hour data sets of EC from passages 4 and 5. Differences at 24 hours did not reach statistical significance. **e**)** Manual FVIII:c assay standard curve**. Ln FVIII, logarithm of FVIIIc activity (U/ml). Note clotting time in samples exceeded 240 seconds.

FVIII expression was next examined on the surface of EC using flow cytometry. HPAEC and HPMEC, but not HUVEC demonstrated significant surface expression of FVIII compared to control untreated (Figure 4a) and isotype control-treated (Figure 4bi) cells from the same well. When compared to the isotype control-treated HPAEC, the overall increase in RFI was shown to reflect stronger staining of a small proportion of EC (Figure 4bii, Figure 4biii). Expression of vWF on the cell surface of a proportion of HPAEC was also detected (Figure 4c).

### FVIII/vWF Secretion

Conditioned media from HPAEC and HPMEC, but not HUVEC detected higher (low mU) levels of FVIII than respective control media (Figure 4d). Overall, approximately 5 mU/mL FVIII accumulated over 48 hours in the conditioned media from a pulmonary EC surface area of 3.8 cm^2^. There was no significant difference in the quantity of FVIII secreted into conditioned medium supernatants following 24 or 48 hour treatment of HPAEC or HPMEC with thrombin (individual *p* values >0.11, data not shown). In addition, conditioned media from HPMEC also had significantly higher mean levels of vWF antigen after 48 hours incubation (1.40 [SD 0.20] U/ml) compared to 24 hours (0.74 [SD 0.058] U/ml).

To test whether the FVIII antigen was functional, manual FVIIIc assays were performed. Standard curves were generated using replicate serial dilutions of lyophilised FVIII concentrate representing a range of 0.1% (0.001 U/ml) to 100% (1.0 U/ml). Clotting times ranged from 58 seconds (1.0 U/ml FVIII) to 120 seconds (0.001 U/ml VIII), with a good standard curve (r^2^ = 0.993, Figure 4e). In contrast, clotting times exceeded 240 seconds in all conditioned media samples, and no clot formation was observed, even at 30 minutes. Diluted test samples were also assayed using the automated CA7000 FVIIIc assay, but again, no clot was detected in any sample.

### Multiple FVIII Transcripts in Pulmonary EC

To confirm that FVIII was synthesised in endothelial cells, polyadenylated FVIII transcripts were analysed by reverse transcription (rt) using an oligo-dT primer and amplification of cDNA products by specific polymerase chain reactions (PCR).

**Figure 5:**
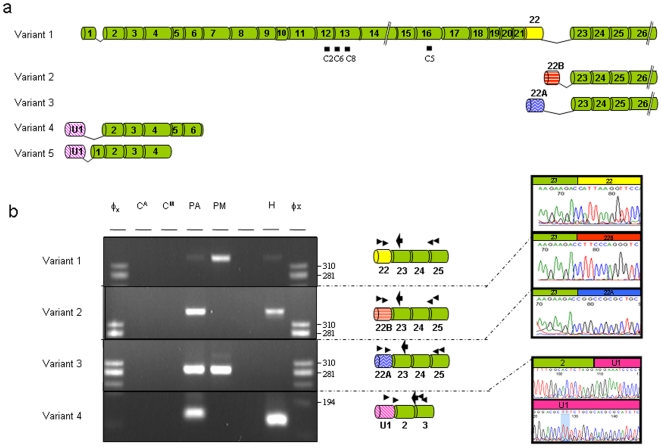
FVIII splice isoforms. **a: Variants identified by ExonMine**: Variants 1 and 2 correspond to major and minor RefSeq isoforms; variants 3–5 to expressed sequence tag (EST) sequences deposited in Genbank. Note none of the alternate variants encode the FVIII mAb epitopes. **b: Variants identified in EC**. Simultaneous expression of variants 1–4 in HPAEC (PA), HPMEC (PM), and HUVEC (H). Gels: φx, HaeIII-digested φx marker, C^A^ negative water control for HPAEC/HUVEC, C^M^ negative water control for HPMEC. The apparent difference in size of variant 4 is an artefact due to gel running (note differential site of 194 marker band in first and last lanes). Cartoons: Thin and thick arrows indicate sites of PCR and sequencing oligonucleotide primers respectively. Sequence chromatograms were obtained using nested reverse internal primers in exon 23 (variants 1–3) or exon 3 (variant 4; low concentration first round product sequenced). Note V5 sequences (exons U1-1-2-3) were not amplified from EC in any reaction.

In nested rt-PCR reactions, in addition to full length FVIII transcript (variant 1), three further transcripts were detected, the known RefSeq variant 2 and two new transcripts (Figure 5, see also exon positions in Figure 1). Variant 2, and novel variant 3 utilised different first exons (22B and 22A) within intron 22, splicing to constitutive exon 23. Novel variant 4 had a first exon (U1) derived from genomic DNA 4.3 kb 5′ to the first exon of the full length transcript, variant 1. All four transcripts were also detected in dermal microvascular EC, and for all EC, FVIII transcripts were detected more readily in equivalent concentrations of cDNA derived from confluent EC (data not shown). The four alternate first exons contained AUG start codons in-frame with the open reading frame in subsequent exons. Between different cell types, there was a possible reciprocal relationship between expression of exon U1 (and exon 22B)-containing transcripts with full length isoform sequence (Figure 5b). Exon 22A sequences amplified preferentially from pulmonary EC (Figure 5b).

**Figure 6:**
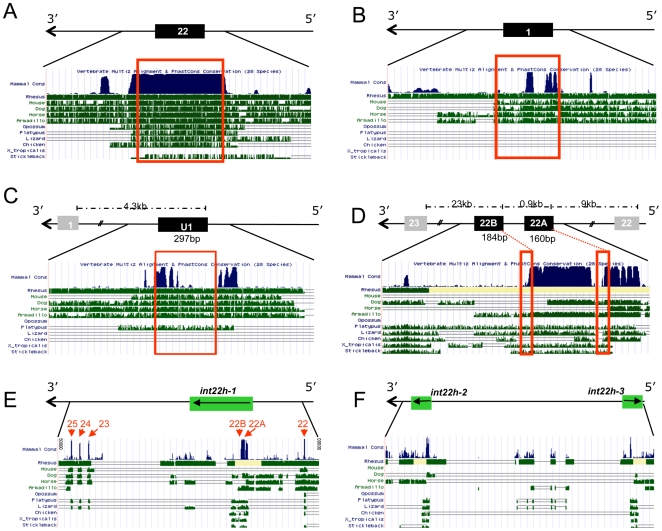
Evolutionary conservation of alternatively spliced exons. **a–d**)**:** Sequence conservation on the UCSC Genome Browser for sequences flanking **a**) exon 22 encoding part of the FVIII light chain; **b**) exon 1, **c**) alternate first exon U1, and **d**) alternate first exons 22A and 22B. Note that the FVIII gene is on the complementary strand, hence 5′ to 3′ is represented right to left. Dotted lines above the exons represent genomic DNA distances; red boxes denote exact sites of exons. **e,f**)**:** Sequence conservation spanning *int22h* repeats in **e**) intron 22 (*int22h-1*, note positions of exons 22A and 22B), and **f**) telomeric X chromosome repeats *int22h-2* and *int22h-3*.

Coding transcripts of importance are subject to strong selective pressure, as demonstrated by the strong evolutionary conservation of exon 22 sequences encoding part of the FVIII light chain (Figure 6a). The most striking feature of the alternate exons U1, 22A and 22B was the degree of conservation of 5′ flanking sequences, compared to those for exon 1, that are part of the characterised FVIII promoter region [32], [33] (Figure 6b, Figure 6c, Figure 6d). Exons 22A and 22B are sited within the *int22h-1* repeat which shares significant homology with the *int22h-2* and *int22h-3* sequences also on the X chromosome (Figure 6e, Figure 6f). Nevertheless, BLASTn alignments confirmed that the novel exon sequences were identified only once in human genomic DNA sequences.

## Discussion

In this study we report that endothelial cells, particularly pulmonary endothelial cells, are a source of FVIII synthesis, with evidence of cell surface expression, secretion, and co-expression with vWF. The study also highlights the complex alternative splicing patterns employed by the FVIII gene in endothelial cells.

Limitations of the study include the relatively limited number of donors examined, particularly for a protein with plasma concentrations that can differ three-fold and remain within normal laboratory ranges (45–158 iu/dl at our institution). Nevertheless, in view of the potential implications of the findings for haematological and pulmonary vascular disorders, we believe it is important to bring our results to the attention of the wider biomedical communities at this stage.

The study demonstrates that endothelial cells from several vascular beds in addition to those of the hepatic sinusoid [13], and pulmonary microvascular circulation [19], can synthesise and secrete FVIII. PubMed searches suggest apparently similar findings were reported for ‘FVIII antigen’ in human umbilical vein EC [34], [35], but it is important to note that this work referred to an antigen that was present at normal levels in haemophiliac plasma, but at reduced levels in plasma from patients with von Willebrand's disease [35], representing what we would now term VWF:Ag, as described in [36]. In our hands, HUVEC were not a good source of FVIII, and indeed were used as a negative control for ELISA and FACS analyses.

Secreted FVIII would be unstable unless associated with vWF. Weibel Palade bodies, now recognised as VWF storage bodies, were first described in pulmonary endothelial cells [31]. This study demonstrates not only that endogenous vWF and FVIII can be secreted and expressed by the same endothelial cells, but also, that in some pulmonary artery EC, endogenous FVIII colocalises with vWF-containing Weibel Palade bodies (Figure 4e, Figure 4f, Figure 4g). Further support for this localisation is obtained from the studies by others that have demonstrated recombinant FVIII may localise within Weibel Palade bodies [37], [38]. The FVIII released into conditioned media in our experiments did not exhibit procoagulant activity. Since even completely FVIII-deficient plasma has an APTT less than 240 s, and others [19] detected FVIII:c activity in pulmonary microvascular EC conditioned media, we assume that particular media conditions may contain inhibitors or proteolytic inhibitors. In addition, it is possible that not all FVIII:Ag produced by pulmonary endothelial cells is active, suggesting it may be important to explore further which specific factors activate any latent antigen in *vivo*, in different experimental media.

While general endothelial expression of FVIII:Ag is clearly important, it is the particular contribution of the pulmonary endothelial bed which carries greater potential to influence general coagulation pathways: The pulmonary endothelial cell–blood interface area exceeds 60 m^2^ [18]: Were *in vitro* accumulation rates to be replicated from pulmonary EC *in vivo*, this would approximate to constitutive secretion of FVIII:Ag of 1×10^6^ mU/hr; or 50 mU per cardiac cycle. Taken together, these observations suggest that the pulmonary endothelium could be one source of the releasable pool of FVIII that can be secreted with vWF, as predicted from clinical studies [39].

The potential for dysregulated release of FVIII by the pulmonary endothelium may carry implications for two forms of pulmonary hypertension. In chronic thromboembolic pulmonary hypertension, 50% of patients have no evidence of a preceding venous thrombus, leading to the concept that a primary pulmonary arteriopathy and *in situ* thromboses could be causal or contributory factors [40], [41]. In primary pulmonary artery hypertension, microvascular intrapulmonary thromboses are recognised as part of the pathological spectrum. Both of these pulmonary hypertension phenotypes are associated with elevated total plasma levels of FVIII and vWF. The current findings that a proportion of pulmonary EC express FVIII and vWF on the cell surface, together with recent evidence that resting EC can constitutively express membrane constituents involved in sustained FVIII/IXa-dependent activation of FX [42], lead us to hypothesise that dysregulated pulmonary endothelial FVIII processing may contribute to exuberant local intravascular thrombus formation in these pulmonary hypertension phenotypes. Further work on tissues from patients with these pathologies is required to support or refute this hypothesis.

A consistent feature of all experiments in this study was that adjacent EC could express different levels of FVIII. We hypothesised that this variation, and potentially the low level steady state FVIII full length transcript mRNA levels and other recombinant FVIII expression limitations observed by others [11], [21], [43], may be due to expression of different splice isoforms of FVIII by different cells. The current study provides evidence for four alternate transcripts of FVIII in the endothelium. Noting that alternate splicing and alternate transcription initiation are recognised to provide major regulatory potential for higher organisms [44], we considered whether the alternate FVIII splice isoforms were likely to be important.

All three short transcripts have the potential to encode short segments of FVIII protein sequence. While direct contribution of any short transcript-encoded protein to FVIII catalytic activity would be unlikely in view of the exons involved (Figure 1, Figure 6), roles for such proteins facilitating or interfering with the complex intermolecular associations required to generate activated FVIII could be postulated. Alternatively, the option for alternative transcript initiation may provide a further layer of regulatory control: The short transcripts do not contain the sequences previously implicated in FVIII transcriptional silencing [45], but it is recognised that minigene insertion into FVIII intron 1 improves *in vivo* production of FVIII [46]. Our data suggest that further examination of whether *trans* expression of 22A-containing transcripts facilitates FVIII processing, or whether switching to U1- and 22B-containing transcripts limits full length FVIII transcript production, may be informative.

Genome-wide analyses indicate that alternatively spliced sequences expressed differentially between tissues are subject to strong selective pressure [47], and there is clearly strong conservation of sequences flanking the alternatively spliced first exons U1, 22A and 22B. This suggests that these 5′ flanking sequences, sited up to 140kb from the characterised FVIII promoter, may have functional roles, and that further evaluation of promoter activity within these regions would be appropriate. Other factors however, may be driving the evolutionary conservation in these regions: First, there are genes on the opposite strands of exon U1 (*FUNDC2*) and exons 22A and 22B (*H2AFB3*). Second, exons 22A and 22B lie within the 9.5kb *int22h-1* sequence which undergoes intra-chromosomal gene conversion with the repeats *int22h-2* and *int22h-3* [48]. This region is well known to haematologists, since homologous recombination between *int 22h-1* and one of the telomeric repeats on the X chromosome leads to the intron 22 inversion responsible for 40% of severe haemophilia.

In summary, this study provides further evidence for endothelial cell sources of FVIII expression of potential importance to haematological disease states and pulmonary vascular thromboregulation. Detailed evaluation of pathways governing FVIII:Ag/FVIII:c relationships, and co-transcriptional modulation of alternate FVIII splicing and transcription initiation by cell type or exogenous stimuli may be required for a better understanding of the molecular mechanisms regulating cell surface and plasma levels of FVIII.
